# Cholesterol-Lowering Phytochemicals: Targeting the Mevalonate Pathway for Anticancer Interventions

**DOI:** 10.3389/fgene.2022.841639

**Published:** 2022-03-22

**Authors:** Kagiso Laka, Lilian Makgoo, Zukile Mbita

**Affiliations:** Department of Biochemistry, Microbiology and Biotechnology, University of Limpopo, Sovenga, South Africa

**Keywords:** phytochemicals, cholesterol, mevalonate pathway, cancer, p53

## Abstract

There are a plethora of cancer causes and the road to fully understanding the carcinogenesis process remains a dream that keeps changing. However, a list of role players that are implicated in the carcinogens process is getting lengthier. Cholesterol is known as bad sterol that is heavily linked with cardiovascular diseases; however, it is also comprehensively associated with carcinogenesis. There is an extensive list of strategies that have been used to lower cholesterol; nevertheless, the need to find better and effective strategies remains vastly important. The role played by cholesterol in the induction of the carcinogenesis process has attracted huge interest in recent years. Phytochemicals can be dubbed as magic tramp cards that humans could exploit for lowering cancer-causing cholesterol. Additionally, the mechanisms that are regulated by phytochemicals can be targeted for anticancer drug development. One of the key role players in cancer development and suppression, Tumour Protein 53 (TP53), is crucial in regulating the biogenesis of cholesterol and is targeted by several phytochemicals. This minireview covers the role of p53 in the mevalonate pathway and how bioactive phytochemicals target the mevalonate pathway and promote p53-dependent anticancer activities.

## Introduction

The mevalonate pathway has been extensively studied in relation to its role in the cholesterol synthesis and its implications for cardiovascular diseases, but in recent years, it has emerged as a challenging and, at the same time, fascinating topic, as several experimental and clinical studies suggested that inhibiting non-sterol isoprenoids could have therapeutic benefits (Yeh et al., 2018). Research studies have shown that the mevalonate pathway is a fundamental regulator of tumorigenesis, with therapeutic implications (Brennan et al., 2010; Clendening et al., 2010; Abate et al., 2017; de Wolf et al., 2017; Göbel et al., 2020a). In addition to controlling cholesterol production, this pathway also affects posttranslational modifications (such as isoprenylation) of Rho-GTPases, which are both associated with tumour progression. Mevalonate pathway inhibitors have been shown to reverse tumorigenesis, suggesting that this pathway is an attractive target for novel therapeutics (Göbel et al., 2020b). Numerous phytochemicals exert effects on cholesterol metabolism, and their cholesterol-lowering properties have attracted the interest of researchers, worldwide (Leng et al., 2018).

Phytochemicals are secondary metabolites, which are largely found in plants and fruits, are associated with preventive properties against several human diseases and their management (Leitzmann, 2016). They are incorporated into our daily diet through the intake of fruits, vegetables, grains and tea. Therefore, they are nutritional constituents in our foods with health benefits. These health benefits have been demonstrated to alleviate a major of chronic diseases that include cardiovascular diseases (Blekkenhorst et al., 2018), diabetes (Sluijs et al., 2015), osteoporosis (Wei et al., 2012) and cancer (Leenders et al., 2014). These secondary metabolites have also been demonstrated to possess anti-microbial, anti-inflammatory, antioxidant, immunomodulatory, detoxifying, and neuropharmacological agents (Kris-Etherton et al., 2002; Shanley and Luz, 2003). The major classes of phytochemicals include polyphenols, terpenoids, and steroids, alkaloids, tannins, saponins, and thiols (Ali Ghasemzadeh and Ghasemzadeh, 2011; Zhang et al., 2015). All these types of phytochemicals are credited with all the mentioned health benefits and it is critical to further understand their role in therapeutics.

Most importantly, phytochemicals are known to act against reactive oxygen species (ROS) that are linked to a plethora of chronic diseases, including cancer. Most phytochemicals exert their preventative action by neutralizing ROS. Elevation of ROS has been attributed to cholesterol elevation, which by this virtue, are linked to carcinogenesis (Leenders et al., 2014). Several phytochemicals have been shown to exert their beneficial effects by lowering circulating cholesterol levels or preventing lipid oxidation, while other phytochemicals have anti-inflammatory and antiplatelet activities (Upadhyay and Dixit, 20152015). *Raphanus sativus* (black radish) juice has been proven to reduce plasma cholesterol levels in mice and dissolve gallstones (Castro-Torres et al., 2014). Glucosinolates are the main secondary metabolites of black radish, which can be hydrolysed into their respective isothiocyanates, and have shown antioxidant properties and the ability to lower liver cholesterol levels by inhibiting the expression of enzymes and transcription factors associated with cholesterol metabolism (Castro-Torres et al., 2014). Furthermore, dietary saponins can directly inhibit the absorption of cholesterol in the small intestine or indirectly inhibit the reabsorption of bile acids to lower plasma cholesterol. Additionally, phytochemicals have been shown to decrease the level of low-density lipoprotein (LDL), which signals cholesterol build-up, thus indicating that phytochemicals can also be used to reduce blood cholesterol levels, thus preventing the accumulation of unwanted cholesterol (Suido et al., 2002; Maheshwari, 2020). The reduction of cholesterol in the enterohepatic circulation leads to cholesterol synthesis being stimulated mostly by the liver. In certain foodstuffs, naturally occurring compounds, such as tocotrienols, may suppress hepatic cholesterologenesis. Such foodstuffs could be combined with saponin-containing foods to control hypercholesterolemia more effectively, since saponins from natural food are risk-free. Additionally, flavonoids, phytosterols, phenolics and alkaloids have been reported to play an important role in human health and the prevention of chronic disease by lowering cholesterol (Ma et al., 2017; Moreau et al., 2018; Adeleke and Babalola, 2020). The regulation of cholesterol homeostasis is now receiving a lot of attention, especially its role in carcinogenesis, and potential therapeutic interventions.

## The Nature of Cholesterol

Cholesterol is a type of lipid that is required to build cells, make vitamins and other hormones such as testosterone and oestrogen (Zampelas and Magriplis, 2019), and is generally absent in prokaryotic cell membranes. Cholesterol has a special ability to upsurge the order of lipids in fluid membranes while maintaining fluidity and diffusion rate. Cholesterol imparts a low permeability barrier to lipid membranes and performs a crucial role in controlling the mammalian cell membrane properties (Moreau et al., 2018). Cholesterol is synthesized *de novo* in the liver and intestines, without omitting the fact that it is obtained from the diet (Zampelas and Magriplis, 2019). The synthesis and utilization of cholesterol must be tightly regulated to avoid its over-accumulation and abnormal deposition within the body, which is linked to various diseases such as cardiovascular disease (Kratzer et al., 2014), stroke (Marijana et al., 2013), Huntington disease (Valenza et al., 2005), and cancer (Lin et al., 2015). The synthesis and utilization of cholesterol are regulated in normal cells; however, in cancer cells, cholesterol biosynthesis is mostly in excess (Chen and Hughes-Fulford, 2001; Cruz et al., 2013). To compensate the increased cholesterol uptake and synthesis, a variety of cancer cells adjust their cellular mechanisms by increasing the expression of cholesterol acyltransferase 1 (ACAT1). In addition to converting cholesterol into cholesterol ester, this enzyme may act as a supply of cholesterol ester for rapid cell division (Heo et al., 2018).

In order for cells and the body to function properly, cholesterol homeostasis is essential. In addition to cardiovascular disease, an increased number of neurological and cancer-related diseases are linked to disturbed cholesterol balance (Luo et al., 2020). Cancer cells tend to have high cholesterol levels, although its significance is questionable (Smith and Land, 2012; Krycer and Brown, 2013). A few epidemiologic studies indicate that elevated serum cholesterol levels increase the risk of prostate cancer (Pelton et al., 2012; Shafique et al., 2012; Allott et al., 2014).

A growing body of evidence indicates that glycolytic activity is elevated in various types of cancer, a phenomenon commonly known as the Warburg effect, which is an essential characteristic of the cancer phenotype (Fantin et al., 2006; Weinberg et al., 2010; Bensinger and Christofk, 2012). There has been long debate about whether elevated cholesterol leads to malignant cell transformation, with previous work suggesting that cholesterol injections into tumour xenografts accelerated tumour growth (Robertson and Burnett, 1913). A causal link between multistep oncogenesis and cholesterol homeostasis has never been proven genetically; however, among its functions, cholesterol is essential for membrane biogenesis (Castoreno et al., 2005), cell proliferation (Fernández et al., 2005; Sun et al., 2014), and differentiation (Liu et al., 2020; Trindade et al., 2021). Researchers continue to study the role of cholesterol in cancer development and the regulation of cholesterol synthesis (Ginestier et al., 2012; Krycer et al., 2012; Haque et al., 2018; Mustra Rakic et al., 2019; Sun et al., 2021).

Phytochemicals have shown plenty of benefits towards the regulation of cholesterol metabolism, and thus, reducing cancer risk. Several phytochemicals have been shown to modulate p53, thus, inducing anticancer signalling pathways, including p53-dependent apoptosis. There are several anticancer mechanisms that are p53-dependent, and phytochemicals also target p53-dependent reduction of cholesterol to reduce cancer risk (Heo et al., 2018; Fang et al., 2019; Allegra et al., 2020). There are several ways that p53 suppresses tumorigenesis, which include the metabolic pathways and one of these includes the regulation of the mevalonate pathway (Freed-Pastor et al., 2012).

## The Role of p53 in Cholesterol Biosynthesis

Cholesterol is synthesized via a cascade of enzymatic reactions within the cytosol and endoplasmic reticulum (ER), which are collectively known as the mevalonate (MVA) pathway. The mechanism behind the synthesis of cholesterol has been well documented (Ayyagari et al., 2020). There is growing evidence that suggests a critical role played by p53 in cholesterol metabolism and tumour suppression (Moon et al., 2019).

Statins were demonstrated to selectively lower cholesterol in hepatoma cells, inducing p53-dependent apoptosis in cancer cells, sparing the primary hepatocytes (Kah et al., 2012).Kah et al. (2012) demonstrated that potent knockdown of the wild-type p53 in Huh7 cells, which is overexpressed, restored the cells’ sensitivity to statin-induced toxicity. Statins show antitumour effects independent of cholesterol production and can be boosted by mevalonate or geranylgeranyl pyrophosphate supplementation, prerequisites for prenylation of small G proteins (Kah et al., 2012). Several types of cancers have been shown to be suppressed through p53-dependent inhibition of the mevalonate pathway (Moon et al., 2019). It is important to note that the mevalonate pathway is differentially regulated by mutant and wild-type p53 proteins. The mutant-p53 forms an association with Sterol regulatory element-binding protein 2 (SREBP-2) at the promoters of the latter’s regulated genes involved in the mevalonate pathway (Freed-Pastor et al., 2012); however, the wild-type p53 represses transcriptional activity of SREBP-2, acting as a master transcriptional regulator of the *ATP-binding cassette transporter* gene (*ABCA1*) (Yamauchi et al., 2015). This proves the importance of the mevalonate pathway in cancer since both versions of p53 intersect with SREBP-2.

In a mouse model of liver cancer in an *in vivo* study, p53 downregulated expression of mevalonate-involved gene products, which included *Mevalonate Kinase* (*MVK*), *farnesyl-diphosphate farnesyltransferase 1* (*FDFT1*) and *3-hydroxy-3-methylglutaryl-CoA reductase* (*HMGCR*) in premalignant hepatocytes, thus suppressing tumour growth. Additionally, pharmacological inhibition of the mevalonate pathway or RNA interference prevents the development of p53-driven murine hepatocellular carcinomas. Similarly, to loss of p53 function, the loss of *ABCA1* increases SREBP-2 maturation in the murine liver, promoting tumour development. Moon et al. (2019) concluded that repression of mevalonate metabolism plays a critical role in preventing liver tumours via p53-mediated mechanisms. Moon and colleagues (Moon et al., 2019) activated endogenous wild-type p53 in different cells to determine whether p53 might downregulate the mevalonate pathway. In human hepatic adenocarcinoma (SK-HEP-1) cells, mouse embryonic fibroblasts and human colon cancer (HCT116) cells treated with Nutlin-3, a p53 activator, the expressions of several genes in the mevalonate pathway were markedly suppressed. The mevalonate pathway-associated genes (*MVK*, *HMGCR*, *Isopentenyl-Diphosphate Delta Isomerase 1* (*IDI1*) and *mevalonate 5-diphosphate decarboxylase* [*MVD*]) were also inhibited by re-activating wild-type p53 at the permissive temperature in hepatocellular carcinoma (Hep3b) cells that were stably expressing a temperature-sensitive mutant-p53 (Friedman et al., 1997). The p53 has been shown to guard against mevalonate-linked tumorigenesis, and its anticancer activity of suppressing SREBP-2 is compromised by inactivating p53 mutations (Yamauchi et al., 2015). The p53 is also involved in the induction of senescence, which has been demonstrated to suppress progression prostate cancer progression (Zhang et al., 2018). Furthermore, mevalonate pathway has been documented to be inhibited via senescence induction in glioma cells expressing a wild-type p53 (Wei et al., 2020). This further supports the involvement of mevalonate in the carcinogenesis process and the need to further target it for therapeutic interventions.

**FIGURE 1:**
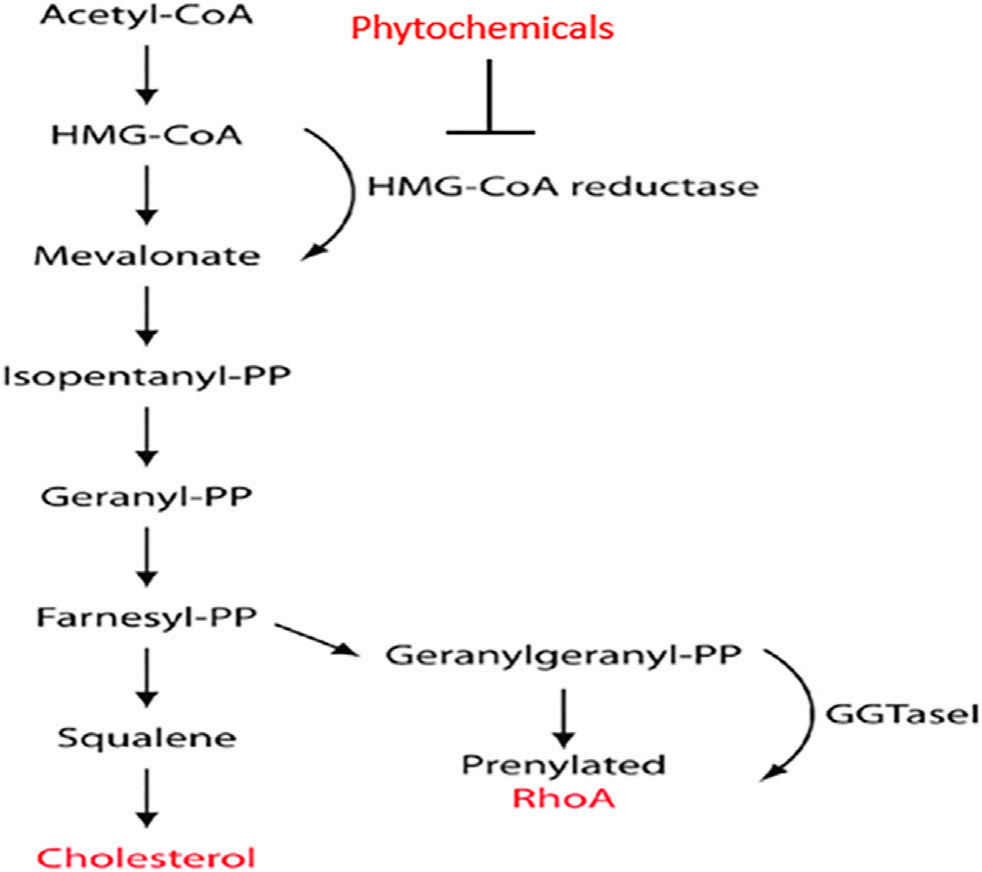
Overview of the mevalonate pathway. The diagram includes the most crucial enzymes, the mediators of synthesis and the point at which phytochemicals disturb the metabolism of HMG-CoA (Hissa and Pontes, 2018).

More reports are emerging, which show that targeting the rate-limiting mevalonate enzyme, 3-hydroxy-3-methyl-glutaryl reductase (HMGCR) for anticancer intervention is a promising strategy (de Wolf et al., 2017; Luttman et al., 2021). *HMGCR* is often referred to as an oncogene and is highly expressed in several cancers, especially those that are p53-deficient. It has been reported that inhibition of HMGCR is critical for apoptosis induction in cancer cells (de Wolf et al., 2017; Luttman et al., 2021). Studies targeting cancer cells revealed that cholesterol synthesis is enhanced in tumours, compared to untransformed cells (Ding et al., 2019). There is a heavy reliance on statins to reduce cholesterol levels; however, there are several phytochemicals that have shown a similar action (Leng et al., 2018). Taken together, these findings validate the importance of the mevalonate pathway in cancer metabolism. This pathway begins with acetyl-CoA, which is derived from an oxidation reaction in the mitochondria (Figure 1). Acetyl- CoA is used to synthesize 3-hydroxy-3-methylglutaryl coenzyme A. HMG-CoA resides in the membrane of the ER and catalyses the rate-limiting reduction of HMG-CoA to mevalonate (Biswas et al., 2012). This is an important regulatory step in the cholesterol synthesis pathway, which is commonly used as a therapeutic target using lipid-lowering drugs such as statins (Teicher et al., 2012).

Cholesterol plays a significant role in cell membrane synthesis, cell growth and cell differentiation (Lin et al., 2008; Cecchi et al., 2009). During the life of an animal, cholesterol is synthesized from acetate precursors or taken up from exogenous and dietary sources. It has been established that bile acids are the major vehicles for the catabolic clearance of cholesterol (Spady et al., 1986). The regulation and maintenance of cholesterol homeostasis are closely influenced and controlled by various feedback mechanisms.

In cholesterol biosynthesis, cholesterol 3-hydroxy-3-methylglutaryl coenzyme A (HMG-CoA) reductase is the target of the feedback mechanism, while low-density lipoprotein (LDL) receptors are involved in cholesterol uptake, and cholesterol 7α-hydroxy-lase is involved in cholesterol metabolism (Russell, 1992). During cholesterol uptake, 3-hydroxy-3-(LDL) receptors and cholesterol 7α-hydroxylase play a critical regulatory role in cholesterol catabolism, while methylglutaryl coenzyme A reductase and the low-density lipoprotein receptor (LDLR) are key in cholesterol synthesis (Russell, 1992). Using lipopolysaccharide (LPS) as a treatment for hepatocellular carcinoma (HCC), He et al. (2017) demonstrated that the increased internal cholesterol concentrations were due to upregulation of LDLR and HMGCR. In addition, the increased cholesterol concentrations promoted the pro-inflammatory state induced by LPS/nuclear factor kappa-light-chain-enhancer of activated B cells (NF-κB).Revilla et al. (2019) found that LDLR expression was up-regulated in more aggressive thyroid tumour cells, while HMGCR expression was down-regulated, increasing cellular cholesterol concentration.Huang et al. (2021) found that high expression of LDLR was associated with poor prognosis in patients with ovarian cancer, whereas high expression of HMGCR was associated with better outcomes.

## Phytochemicals and Their Effect on Cholesterol Synthesis

The liver plays a crucial role in regulating cholesterol metabolism. Cholesterol homeostasis is regulated by several genetic molecules, *including 3-hydroxy-3-methyl-glutaryl-coenzyme A reductase* (*HMGCR*), *cholesterol 7α-hydroxylase* (*CYP7A1*), *ATP-binding cassette transporter* (*ABCA1*), *sterol regulatory element-binding proteins* (*SREBPs*), *Liver X receptor* (*LXR*), and *AMP-activated protein kinase* (*AMPK*). In addition to their uptake, synthesis, intracellular movement, and excretion, these molecules maintain an intricate network (Lee and Tontonoz, 2015; Ness, 2015). HMGCR is responsible for the synthesis of cholesterol (Nyamai et al., 2016). In addition to these functions, ABCA1 participates in the formation of high-density lipoprotein (HDL) and boosts cholesterol oxidation, while SREBP2 controls cholesterol biosynthesis, and activation of LXR leading to increased lipogenesis and fat accumulation. The phytochemicals, which are natural compounds produced by plants for their interactions with the environment, defence against environmental hazards and give them their specific pigments, and aroma (Koche et al., 2016). Phytochemicals such as alkaloids, phenolics, terpenoids, and tannins play an important role in disease prevention (Cao et al., 2012). It is known that several phytochemicals exert effects on cholesterol metabolism, these phytochemicals have attracted interest in cholesterol metabolism due to their cholesterol-lowering properties (Cao et al., 2012) as well as their ability to inhibit intestinal cholesterol absorption (Ikeda et al., 2010). Polyphenols exemplify phytochemicals that inhibit the mevalonate pathway by targeting cholesterol synthesis through gene regulation to lower cholesterol. Li *et al.* (Li et al., 2011) found that mulberry leaf polyphenol extract reduces the protein expression of sterol regulatory element-binding protein 2 (SREBP2) and 3-hydroxy-3-methylglutaryl-CoA (HMG-CoA) in 3T3-L1 cells and obese mice that were fed with a high-fat diet. These results supported another previous study, which also showed the ability of polyphenols to inhibit cholesterol synthesis (Lu and Hwang, 2008). Furthermore, Sun et al. (2018) and Ha et al. (2015) also showed that garlic extract has a similar effect on cholesterol synthesis, garlic and garlic-derived organosulfur compounds reduced the synthesis of cholesterol by inhibiting HMG-CoA.

Statins and amino-bisphosphonates (N-BP) are two major classes of drugs that inhibit the mevalonate pathway at different levels. Low-density lipoprotein receptors can take up extracellular cholesterol in a more efficient manner due to them reducing cholesterol production (Simonetti et al., 2002). For patients with high cholesterol levels, statins are being prescribed as standard treatment (Hadjiphilippou and Ray, 2019). According to epidemiological studies, certain statins may reduce the risk of developing certain types of cancer, such as liver cancer, in patients who take them for cholesterol control (Deng et al., 2018), colorectal (Shi et al., 2014), hepatocellular carcinoma (Thrift et al., 2019), esophageal cancer (Siddiqui et al., 2009), breast (Brown et al., 2016) and ovarian cancer (Shi et al., 2013). Additionally, phytochemicals are also a rising star that targets the mevalonate pathway that is upregulated in some cancers (Castro-Torres et al., 2014).

The anti-oxidative properties of polyphenols, such as catechins, have long been shown to inhibit low-density Lipoprotein (LDL) oxidation (Shi et al., 2003; Couttenier et al., 2017). According to Ngamukote et al. (Ngamukote et al., 2011) gallic acid, catechin, and epicatechin were all found to significantly inhibit pancreatic cholesterol esterase. In a previous study, scientists found that three major polyphenolic compounds present in grape seeds exhibit cholesterol-lowering properties by inhibiting pancreatic cholesterol esterase, binding bile acids, and decreasing the solubility of cholesterol in micelles that delays cholesterol absorption. Similar studies also implicated phytochemicals in lowering the levels of cholesterols in the blood of patients suffering from hypercholesterolemia and other cardiovascular diseases (Castro-Torres et al., 2014). Examples of such phytochemical extracts that can be employed as interventions in reducing blood cholesterol levels include phytosterols (Millar et al., 2017), Flavonoids (Fukumitsu et al., 2010) and Lignans (Harnafi and Amrani, 2007).

Phytochemicals have attracted considerable attention in preventing the onset of many chronic diseases, including cardiovascular failure, cancer, and metabolic disorders. Other compounds exhibit anti-inflammatory and antiplatelet effects, with the ability to reduce circulating cholesterol levels or inhibit lipid oxidation as means of exerting their beneficial effects (Chen et al., 2013). Various fruits, vegetables, and beverages, including teas and wines, contain different types of flavonoids. In addition to reducing platelet aggregation, in the metabolic process of metabolic flux, polyphenolic flavonoids inhibit LDL oxidation reducing cardiac damage from ischemia and reperfusion, reducing plasma cholesterol levels, or reduce coronary artery disease.

Flavonoids are the most studied phytochemicals and are the major secondary metabolites of phenolic plants. Five major classes are found in this family, differing in their chemical composition: flavones, flavonols, flavanones, flavonols and anthocyanidins. A large number of epidemiological and experimental studies have demonstrated the benefit of flavonoids in preventing cardiovascular events (Kim et al., 2016; Wang et al., 2017a; Kim and Je, 2017). In this field, it has been established that a variety of flavonoids found in medicinal plants, fruits, vegetables, spices, and beverages have beneficial effects on parameters related to cardiovascular diseases, most notably hyperlipidemia, hypercholesterolemia, blood platelet aggregation, and vascular reactivity. In terms of lipid metabolic disorders, flavonoids have been found to produce therapeutic effects by reducing total cholesterol, triglycerides, low-density lipoprotein cholesterol (LDL-Cholesterol) and Apolipoprotein B (apoB) levels. The protective effect of these drugs is also accompanied by an increase in HDL cholesterol, bile acid secretion, and lipid catabolism (Tresserra-Rimbau et al., 2014). Also, Harnafi and Amrani, (Harnafi and Amrani, 2007) reported that flavonoids protect against cardiovascular diseases caused by oxidative stress in four distinct ways: direct free radical scavenging, chelation of pro-oxidant metal ions, and inactivation of LDL oxidation by an antioxidant-rich HDL complex retaining HDL-associated paraoxonase activity. Furthermore, flavonoids have the ability to inhibit enzymes involved in oxidizing LDL particles, such as phospholipase A2, cyclooxygenase and lipoxygenase, glutathione reductase, xanthine oxidase and nicotinamide adenine dinucleotide phosphate (NADPH)-oxidase (Tresserra-Rimbau et al., 2014). *Hibiscus sabdariffa* leaf polyphenolic extract, a flavonoid-rich extract, has been shown to prevent foam cell formation and intracellular lipid accumulation in oxidised-LDL (ox-LDL)-induced macrophages and increased Liver X receptor alpha (LXRα)/ABCA1 activity, which inhibited atherosclerosis and stimulated cholesterol removal from macrophages (Leng et al., 2018).

A liver x receptor (LXR) acts as a “cholesterol sensor”, working in a similar way to sterol response element binding proteins (SREBPs), lowering cholesterol levels by increasing RNA expression of target genes associated with reverse cholesterol transport, bile acid synthesis, and intestinal cholesterol absorption (Calkin and Tontonoz, 2010; Piccinin et al., 2021). Oxysterols, in particular, act as endogenous ligands for LXRs and activate transcription of target genes (Chen et al., 2007). The LXR activity has been modulated by several natural and synthetic compounds. In addition to their crucial role in cholesterol metabolism, LXRs also play an important role in regulating cancer progression (Komati et al., 2017). Among the oxysterols produced by cholesterol oxidation, there are endogenous oxysterols that are formed either through enzymatic or nonenzymatic reactions, and diet-derived oxysterols (Biasi et al., 2013). The most common oxysterols found in processed and/or stored cholesterol-rich foods are 7α-hydroxycholesterol, 7β-hydroxycholesterol, 7-ketocholesterol, 5α,6α-epoxycholesterol, and 5β,6β-epoxycholesterol (Plat et al., 2005; Kanner, 2007). They possess marked pro-inflammatory and cytotoxic effects in a wide range of cells and tissues and are more readily diffusible through membranes than unoxidized cholesterol (Leonarduzzi et al., 2008; Vejux et al., 2008).

The cholesterol metabolite oxysterols have been shown to have pro-apoptotic effects on colon cancer cell lines (Roussi et al., 2005; Biasi et al., 2013). A major oxysterol found in the plasma may function as an LXR ligand, as 27-hydroxycholesterol (27-OHC) reduces colon cancer cell proliferation (Warns et al., 2018). It is interesting to observe that the protein levels of LXRα and its target gene, *ABCA1*, are downregulated in primary colon cancer specimens and in synchronous liver metastases (Lo Sasso et al., 2013). Furthermore, both LXRα and liver X receptor β (LXRβ) are downregulated in a cohort of colorectal cancer patients, which is associated with an increase in cholesterol levels (Sharma and Agnihotri, 2019). In general, these data indicate a role for LXR activation in colorectal cancer-mediated antitumor effects.

Several types of cancer have been investigated for the role of LXR in carcinogenesis, including glioblastomas, melanoma, breast cancers, and lung cancers (Guo et al., 2011; Nelson et al., 2013; Pencheva et al., 2014; Dai et al., 2016). It is noteworthy that LXR agonist treatment *in vivo* elinimate glioblastoma cancer cells in a cholesterol-dependent manner, leading to tumour regression and prolonged survival (Villa et al., 2016). Following transcriptional stimulation by SREBP2, genes necessary for cholesterol biosynthesis and uptake are activated. The intermediate precursors in cholesterol biosynthesis, oxysterols and desmosterols, stimulate their cognate receptors, LXRs, when cholesterol levels increase in the cell. The LXR-mediated increase in transcription further increases cholesterol efflux by enhancing the transcription of transporters such as ATP-binding cassette A1 (ABCA1) and ATP-binding cassette G1 (ABCG1) that are responsible for ferrying excess cholesterol to high density lipoproteins (HDLs). When an HDL particle detects the scavenger receptor type B, class-I (SR-B1) in the liver, it delivers its cargo into the bile, which is primarily responsible for removing excess cholesterol (Ramírez-Jiménez et al., 2015; Linton et al., 2017; Sharma et al., 2019).

As demonstrated in an *in vitro* study and *in vivo* assays with C57BL/6 mice, extracts of common beans rich in flavonoids and saponins influenced lipid metabolism by increasing biliary excretion of cholesterol and modulating the liver X receptor (LXR), which is known to regulate cholesterol and fatty acid metabolism by reducing adenosine monophosphate-activated protein kinase (AMPK) [124]. In THP-1 macrophage-derived foam cells, leonurine promoted cholesterol efflux and alleviated cellular lipid accumulation in response to the PPARγ/LXRα signalling pathway, and it prevented atherosclerosis in mice deficient in apoE−/− mice (Jiang et al., 2017). Further, Lin et al. (Lin et al., 2017) demonstrated that allicin-induced upregulation of ABCA1 promoted cholesterol efflux and reduced lipid accumulation through PPARγ/LXRα signalling in macrophage-derived foam cells. Three isolates (stigmast-4-en-3-one, cabraleahydroxylactone 3-acetate, and cabraleahydroxylactone 3-acetate) from the dichloromethane fraction of methanol extract of *Dysoxylum tpongense* plant possessed an anti-inflammatory effect against Liver X receptor (LXR) activation in HepG2 cell line (Pham et al., 2021).

There are over 100 different phytosterol types, which are naturally occurring substances derived from plants and found in nuts, vegetable oils, seeds, cereals, and legumes. Their structures and functions are very similar to those of vertebrate cholesterol (Zhou et al., 2012).

It is well established that plant sterols significantly lower low-density lipoprotein (LDL) cholesterol levels, and products containing these compounds have been widely used as dietary approaches to reducing plasma cholesterol and atherosclerosis risk (Chan et al., 2007; Calpe-Berdiel et al., 2009). Plant phytosterols are thought to lower cholesterol in part by competing with intestinal absorption of dietary and biliary cholesterol in mixed micelles (Smet et al., 2012).

The phytosterols that are common in plants are β-sitosterol, campesterol and stigmasterol, which are extremely similar in structure and differ only in the side chains on their molecular backbones (Kritchevsky and Chen, 2005). β-sitosterol and campesterol, are phytosterols, or plant-derived sterols with chemical structure similar to cholesterol. *In vivo* study examined the ameliorative effects of phytorserol (PS), a mixture of 44% β-sitosterol, 26% campesterol, and 19% stigmasterol on LPS-induced lung injury in mice. The results showed that PS administration significantly alleviated LPS-induced lung injury by activating the LXRs/ABCA1 pathway and reducing pulmonary inflammation. He et al. (He et al., 2022) concluded that PS exerts its anti-inflammatory effects via activating LXRs/ABCA1 signalling pathways. Researchers tested the anticancer effects of β-sitosterol and campesterol *in vitro*. Cell viability was significantly reduced by campesterol and β-sitosterol, treatments resulted in DNA fragmentation and apoptosis of the breast carcinoma MCF-7, colon carcinoma HCT116, and cervical carcinoma HeLa cells (Alvarez-Sala et al., 2019). Among the above mentioned phytosterols, β-sitosterol is the most studied in cancer related research. Further studies of β-sitosterol’s antiproliferative effects were conducted on cervical cancer HeLa cells (Cheng et al., 2015), breast cancer MCF-7 (Awad et al., 2007; Rubis et al., 2010) and colon cancer HT-29, Caco-2 and HCT116 (Choi et al., 2003; Awad et al., 2007; Daly et al., 2009; Montserrat-de la Paz et al., 2015).

In a reporter gene assay, fucosterol, a sterol abundant in marine algae, significantly increased LXR transcriptional activity, which was inhibited by arsenic trioxide (As_2_O_3_), a potential antagonist for LXR. Fucosterol, a molecule involved in reverse cholesterol transport, increased the gene expression of *ABCA1*, *ATP binding cassette subfamily G member 1* (*ABCG1*), and *Apolipoprotein E* (*ApoE*), key genes for reverse cholesterol transport, in THP-1-derived macrophages. Additionally, fucosterol was found to regulate intestinal *NPC1 like intracellular cholesterol transporter 1* (*NPC1L1*) and *ABCA1* in Caco-2 cells. In HepG2 cells, fucosterol does not induce cellular triglyceride accumulation, principally because it upregulates *insulin induced gene 2* (*INSIG2*) which delays the nuclear translocation SREBP-1c, a key transcription factor in hepatic lipogenic pathways. According to these data, fucosterol acts as a dual LXR agonist to regulate cholesterol homeostasis in multiple cell lines without inducing hepatic triglyceride accumulation (Hoang et al., 2012). Based on the observations outlined above, in spite of a lack of molecular evidence conclusively indicating the anticancer properties of phytosterols, clinical trials are still necessary to determine their efficacy.

There are several natural antioxidant compounds with anti-obesity properties, including crocin, chlorogenic acid, geniposide, and quercetin, which have been used to treat obesity-related metabolic diseases. A previous study reported a strong synergistic effect of the natural compounds, significantly reducing lipid deposition compared to individual treatments, in both triglyceride and total cholesterol (Jakobisiak and Golab, 2003). Low-density lipoprotein receptor (LDLR) facilitates cholesterol flushing from the liver, but proprotein convertase subtilisin/kexin 9 (PCSK9) inhibits this process by promoting the degradation of LDLR (Kim and Je, 2017). In hepatocytes, the isolated defense compound ((7′E, 8S)-2′,4,8-trihydroxy-3-methoxy-2,4′-epoxy-8,5′-neolign-7′-en-7-one) decreased PCSK9 and activated LDLR (Mbikay et al., 2014). Furthermore, SREBP2 has been found to play an important role in the regulation of PCSK9 and LDLR (Chae et al., 2021).

Organosulfur and phytosterols are among the widely dispersed phytochemicals that have been found to decrease inflammatory signaling molecules and also modulate antioxidant impact by inhibiting NF-κB pathways. An antioxidant derived from lychee fruit, oligonol, inhibited inflammation and oxidative stress through reducing ROS production, lipid peroxidation, and down-regulating NF-kB and inducible nitric oxide synthase (iNOS) expression. Furthermore, 8 weeks of treating type 2 diabetic mice model with oligonol prevented dyslipidaemia, led to reduced levels of SREBP-1 expression, associated with reduced levels of the SREBP-1 target genes *fatty acid synthase* (*FAS*), *acetyl-CoA carboxylase* (*ACC*) and *HMGR* (Noh et al., 2011). NF-kB-p65 is more active in diabetic rats, which indicates a more stressful environment. By inhibiting oxidative–nitrosative stress and pro-inflammatory cytokine production, diosmin significantly inhibited the activation of the NF-kB signalling pathway (Ahmed et al., 2019). Interestingly, these results are in line with those reported in the previous study, which identified diosmin as an inhibitor of NF-kB activation following acute lung injury induced by LPS (Imam et al., 2015). Therefore, these phytochemicals lower total serum and LDL cholesterol levels, thereby protecting the heart from atherosclerosis and cancer (Bloch and Thomson, 1995). There has been great interest in plant-derived compounds that might act as inhibitors of the NF-kB pathway, including lignans (Nam, 2006; Chen et al., 2017).

Lignans form part of the woody components of trees and other plants and are closely related to lignin. Their biological activities and structures range widely. In addition to antitumor, antimitotic, and antiviral properties, lignans have been reported to inhibit a number of specific biological enzymes (Ayres et al., 1990). The traditional use of sesame as a health food has long been extensive; however, this seed’s physiologically active ingredients have not been explored. Among the lignans found in sesame, sesamin is a very intriguing component due to its powerful antioxidant properties (Budowski and Markley, 1951), which are responsible for some of the diseases that can lead to degenerative changes (Glavind et al., 1952; Sevanian and Hochstein, 1985). Sesamin was studied in rats maintained on various dietary regimens to see how it affected various aspects of cholesterol metabolism. As a result of sesamin administration, the liver’s 3-hydroxy-3-rnethylglutaryl coenzyme A reductase activity significantly declined, while the activities of drug-metabolizing enzymes and alcohol dehydrogenase did not change (Fuhrman et al., 2000). Both serum biochemistry and metabolomics studies have shown that *Schisandra chinensis* lignans (SCL) have anti-hyperlipidemic effects. The results showed that the SCL significantly decreased the mRNA expression levels of hepatic lipogenesis genes such as *SREBP-1c*, *fatty acid synthase* (*FAS*), and *acetyl-CoA carboxylase* (*ACC*), and also lowered liver X receptor α (LXRα) expression. The expression levels of SREBP-2 and 3-hydroxy-3-methylglutaryl coenzyme A reductase (HMGCR) in the liver of hyperlipidemic mice were also significantly reduced by SCL (Hirose et al., 1991). Mice treated with *Schisandra chinensis* fruit extract showed significant improvements in insulin resistance, attenuated inflammation, and reduced lipid accumulation in the liver. Screening of different fractions of *S. chinensis* extract revealed that the lignan-rich fraction was the most effective at lowering lipid levels. The major active fraction of *S. chinensis* contained eight abundant lignans; namely, schisandrols A, B and schisandrins B, C, which demonstrated better lipid-lowering effects in human liver cancer (HepG2) cells and schisandrin C showed substantial hypolipidemic effects in hyperlipidemic mice treated with Triton WR-1339 (Sun et al., 2017).

Various epidemiological studies have suggested that dietary lignans have chemopreventive properties, possibly because they act on estrogen, angiogenesis, apoptosis, and oxidation (Webb and McCullough, 2005). Schisandra chinensis lignans have shown antiproliferative effects against a range of human cancer cells. These activities may be influenced by methoxy groups at C-3, C-4, C-3′, and C-4′, hydroxyl groups at C-8′, and the stereo-configuration of the biphenyl ring and angeloyl group. According to additional research, the schizantherin C active compound inhibited the progression of G0/G1 cells in A549 human lung cancer cells (Min et al., 2008). Even the spices we use in our daily foods can reduce cholesterol. In the spice turmeric, curcumin is among the main curcuminoids. It has been extensively studied for its antioxidant, anticancer and anti-inflammatory effects. Curcumin administration has also been shown to lower blood cholesterol levels in various *in vivo* studies (Soni and Kuttan, 1992; Jain et al., 2009). A component of these effects is believed to be through the upregulation of LDL receptors (Shin et al., 2011; Tai et al., 2014).

According to prior *in vivo* studies, curcumin also lowers blood cholesterol levels. Researchers attribute this effect to the upregulation of LDL receptors. However, the uptake of cholesterol from the gut can also influence plasma cholesterol levels, via the particular transporter Niemann-Pick Cl-like 1 (NPC1L1). Curcumin suppresses the expression of NPC1L1 in gastrointestinal Caco-2 cells by inhibiting cholesterol uptake (Peschel et al., 2007; Dou et al., 2008). Curcumin increased the mRNA and protein expression of LDL-R and the subsequent uptake of Low Density Lipoprotein from Human Plasma, DiI complex (DiI-LDL). Using a GFP reporter system in transfected HepG2 cells, the sterol regulatory element of the LDL-R promoter was activated by curcumin. In HepG2 cells, curcumin reversed the Isig2-induced suppression of LDL-R expression. Based on these results, curcumin increases the expression and activity of LDL-R by activating SREBPs (Dou et al., 2008). In a study conducted with a long-term curcumin treatment, hepatic and plasma cholesterol levels were lowered, comparable to the protective effects of lovastatin (Shin et al., 2011). Based on these studies, we gain a deeper understanding of curcumin’s cholesterol-lowering and anti-atherosclerosis effects. In rodents, curcumin lowered the levels of LDL oxidation and cholesterol, thus reducing systemic and tissue-specific inflammation (Aggarwal and Harikumar, 2009). Spices such as ginger are used as medicines due to their effects as rubefactants, diuretics, and stimulants (Giri et al., 1984).

Ginger has been used throughout history for its health benefits and is one of the world’s best-known spices. Monoterpenes and sesquiterpenes are found in the dried ginger extract. It is primarily gingerols and shogaols, as well as related phenolic ketone derivatives that give ginger its antioxidant property (Nemmar et al., 2012). Traditional medicine uses ginger to treat certain disorders, including arthritis, with a variety of pharmacologic effects, including antiemetic, ulcerative, anti-inflammatory, antioxidant, antiplatelet, antidepressant, cholesterol-lowering, cardioprotective, and cancer-preventive effects. (Kazeem et al., 2013; Wang et al., 2017b).

Ginger extract’s anti-atherogenic properties can be attributed to its antioxidative effects on macrophages and plasma total cholesterol (Fuhrman et al., 2000). In response to oxidative stress, macrophages increase their oxidation of LDL, their uptake of oxidized low-density lipoprotein (Ox-LDL), and their peroxidation of lipids (Shi et al., 2013). Despite the absence of transition metal ions, these lipid-peroxidized cells have been found to oxidize LDL (Fuhrman et al., 1994), however, how the process takes place depends on the oxidative state of the LDL (Aviram and Fuhrman, 1998). *In vivo* and *in vitro* experiments have demonstrated that ginger extract decreases macrophage oxidative responses. Previously, Fuhrman et al. (Fuhrman et al., 2000) concluded that ginger extract may be beneficial by preventing the development of atherosclerosis since it is linked with lowered macrophage-mediated oxidation of LDL, reduced uptake of oxidized LDL by macrophages, reduced oxidative state of LDL and reduced LDL aggregation. As a result, cellular cholesterol accumulation and foam cell formation are reduced, both hallmarks of early atherosclerosis.

Cardiovascular diseases are mostly caused by complications related to atherosclerosis, which refers to an inflammatory state caused by an immune response correlated with uncontrolled proliferation of vascular smooth muscle cells, endothelial cells, and macrophages *in situ* (Ouimet, 2013). These two diseases have been linked to nuclear transcription factors such as NF-kB. Newly found angiogenesis modulators have been associated with plaque proliferation and restenosis of atherosclerotic lesions and local and metastatic tumor expansion. Recent advances in atherosclerosis therapeutic strategies include the use of established anticancer treatments, such as growth factor inhibitors and radiation therapy, in an attempt to prevent restenosis after angioplasty and endarterectomy (Ross et al., 2001). There is compelling evidence that atherosclerosis and cancer develop and progress in the same molecular pathways than is previously thought, and that emerging anti-inflammatory and antiproliferative therapeutic strategies may ultimately prove effective for both conditions. Due to the fact that both diseases exhibit multiple etiologies and are multifactorial, they share not only several important molecular pathways, but also a variety of etiological and mechanistical processes from the very earliest stages of development to advanced forms. Genetic alteration, inflammation, uncontrolled cell proliferation, and oxidative stress are important factors contributing to their progression (Tapia-Vieyra et al., 2017).

In mice, ginger significantly lowered cholesterol levels by interfering with intestinal sterol absorption (Fuhrman et al., 2000). A mechanism through which ginger may exert its hypocholesterolemic action is through bile acids (Zhang et al., 2009). Following ginger administration during the initiation and post-initiation stages of colon cancer growth, the levels of free fatty acids, fecal bile acids, HMG CoA reductase, neutral sterols, triglycerides, phospholipase A, tissue cholesterol, and phospholipase C were significantly decreased. Because ginger has hypolipidemic and antioxidative properties, it has been found to reduce the risk of colon cancer considerably (Manju et al., 2006). All these spices are beneficial for the treatment of a plethora of diseases and have been used by traditional healers across the world. A number of phytochemicals have been studied, including leoligin (Scharinger et al., 2016), puerarin (Chung et al., 2008) and geraniol (Galle et al., 2015), which may inhibit cholesterol synthesis by inhibiting HMGCR.

Leontopodium nivale’s roots contain a compound called leoligin, which has shown promising anti-atherosclerotic properties in previous studies. LDL cholesterol levels are reduced by leoligin, and the conversion of LDL to HDL and LDL to total cholesterol ratios are improved (Scharinger et al., 2016). Leoligin has been shown to influence cholesteryl ester transfer protein (CETP), an enzyme that facilitates the transfer of cholesteryl esters from high-density lipoprotein (HDL) to, among other lipoproteins, LDL. Therefore, inhibition of CETP increases HDL cholesterol levels (Barter et al., 2003). Leoligin has also been shown to inhibit CETP in human plasma. The lipid-lowering effect of leoligin on transgenic CETP mice may be due to other mechanisms, as leoligin was given orally even though CETP activity had been measured to be elevated (Reisinger et al., 2009; Duwensee et al., 2011). In addition, leoligin was examined both *in vitro* and *in vivo* in regard to its effects on venous grafts. In a venous bypass graft mouse model, leoligin significantly inhibited intimal hyperplasia in human saphenous veins *in vitro*, and *in vivo*, it inhibited neointimas from forming while causing no endothelial damage. A significant inhibitory effect of leoligin was observed on smooth muscle cell proliferation in primary vascular smooth muscle cells and endothelial cells by inducing cell cycle arrest in the G1 phase as well as inhibition of tumour necrosis factor-alpha (TNF-α)-induced endothelial vascular cell adhesion molecule-1 (VCAM-1) expression (Kou et al., 2016).

Traditional Oriental medicine has used puerarin, an isoflavone glycoside found in the root of the *Pueraria lobata* plant, for thousands of years for various medicinal purposes. Puerarin works by protecting cells from oxidative stress (Fu-Liang et al., 2006). Unfortunately, it is insolubility often prevents it from being bioavailable in humans. The use of natural compounds has been demonstrated to reduce serum cholesterol levels in order to slow down the progression of cardiovascular disease. Despite its multiple effects on hepatic cholesterol metabolism, puerarin and its glycosides caused multiple changes, such as increasing LDL uptake, reducing cholesterol biosynthesis, and possibly enhancing cholesterol degradation. In puerarin glycoside-treated HepG2 cells, LDL receptor promoter activity increased in a dose-dependent manner (Chung et al., 2008) Therefore, HepG2 cells and mouse livers expressed higher levels of LDL receptor mRNA and protein. *In vitro* and *in vivo*, HMG-CoA reductase transcription and translation are down-regulated. In a different study (Ma et al., 2014), a significant increase in Cholesterol 7 alpha-hydroxylase (CYP7A1) mRNA levels was observed in mouse livers. Combined, these studies demonstrate that puerarin and its glycosides are bioactive isoflavones capable of antioxidative and hypocholesterolemic effects in HepG2 cells and inbred mouse strain (C57BL/6J). Several mechanisms are likely involved in puerarin glycoside’s hypocholesterolemic effects on the liver, including increasing LDL uptake, reducing cholesterol synthesis, and possibly enhancing cholesterol degradation (Chung et al., 2008). Puerarin markedly reduced the serum and liver cholesterol levels induced by a hypercholesterolemic diet (Yan et al., 2006). The atherogenic index was reduced significantly as well. A significant increase in the levels of CYP7A1 mRNA expression but not for that of HMGCR and lanosterol 14α -demethylase (CYP51).

Based on these results, puerarin reduced the atherogenic properties of dietary cholesterol in rats. A possible explanation for its hypocholesterolemic function is that it allows cholesterol and bile acids to be excreted by the liver (Yan et al., 2006). Li et al. (Li et al., 2017) demonstrated that puerarin significantly enhanced ABCA1 mRNA and protein expression in human THP-1 macrophage-derived foam cells through activation of AMPK, the peroxisome proliferator-activated receptor gamma (PPARγ-) and LXR-α pathways. By targeting the 3′ untranslated region of serine/threonine kinase 11 (STK11), miR-7 triggered the AMPK pathway. In macrophages treated with puerarin, the miR-7 mimic significantly decreased STK11 expression, decreased AMPK phosphorylation, and decreased PPARγ-LXR-α-ABCA1 expression. Moreover, miR-7 has been shown to decrease cholesterol efflux and increase cholesterol levels in macrophage-derived foam cells from THP-1 macrophages. Based on the results, puerarin seems to improve ABCA1-mediated cholesterol efflux and reduce intracellular cholesterol levels via the pathways involving miR-7, STK11, and AMPK-PPARγ-LXR-α-ABCA1.

The natural isoprenoid geraniol is found in essential oils of several aromatic plants, and it possesses a number of biochemical and pharmacological properties such as antimicrobial activity (Bard et al., 1988) and antitumour activity (Yu et al., 1995; Burke et al., 1997; Chen and Viljoen, 2010; Hosseini et al., 2020). *In vivo*, geraniol was shown to significantly reduce plasma total-cholesterol and triglyceride levels, as well as hepatic fatty acid and lipid biosynthesis. In the liver of mice treated with geraniol, the levels of the protein and activity of HMGCR increased. Additionally, geraniol enhanced the expression of mRNAs that code for LDL and VLDL receptors and decreased the expression of mRNAs that code for *acetyl-CoA carboxylase alpha* (*ACACA*), without altering transcription of the *sterol regulatory element binding transcription factor 2* (*SREBF2*) gene (Zhang et al., 2009).

**FIGURE 2:**
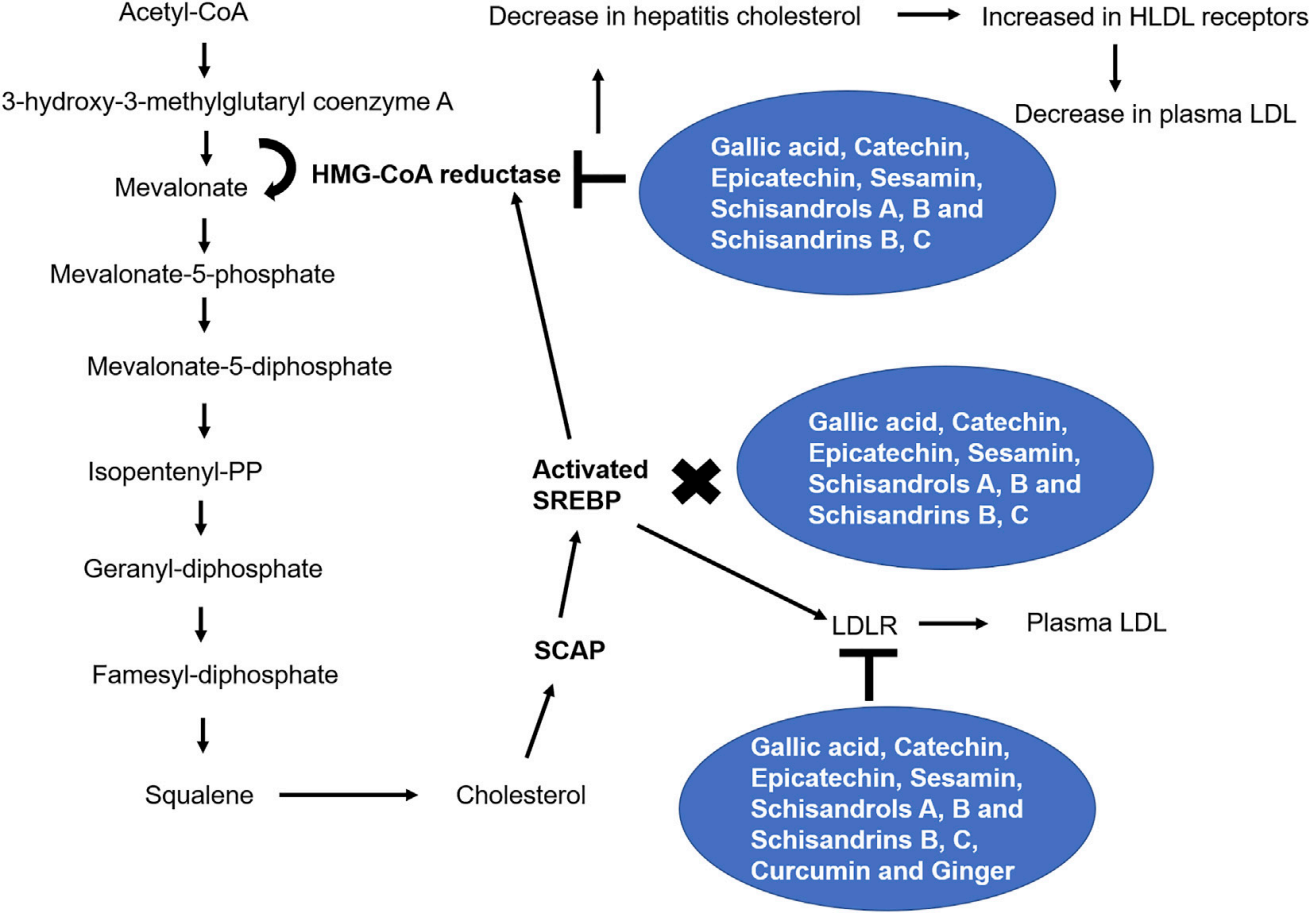
Phytochemicals and the mevalonate pathway. Through several enzymes, glycolysis’ end product, acetyl-CoA, is converted into cholesterol. By inhibiting the mevalonate pathway, statins and secondary metabolites are prevented from translocating to the Golgi apparatus and activating SREBPs (Göbel et al., 2020b).

For more than two thousand years, Chinese physicians have used *Rhizoma coptidis* in food additives, herbal medicine, antibacterial treatment, antiviral therapy, anti-inflammatory treatment, and antihyperglycemic and hypolipidemic treatment (Yi et al., 2013; Kou et al., 2016). Berberine, coptisine, palmatine, epiberberine, and jatrorrhizine, all of which have similar isoquinoline structures, have been widely documented as the primary bioactive compounds of *Rhizoma coptidis* (Chen et al., 2012; Moon et al., 2019). Coptidis alkaloid has been clinically approved as Chinese herbal medicine used as a remedy for dyslipidemia, atherosclerosis, and related diseases because it improves serum lipid profile and has become a patent and clinically approved ingredient of *Rhizoma coptidis* (Hadjiphilippou and Ray, 2019). *Rhizoma coptidis* contains many alkaloids including berberine, coptisine, palmatine, epiberberine, and jatrorrhizine, which improved hamster dyslipidemia in a variety of ways. A combination of the above-mentioned alkaloids demonstrated a greater synergistic cholesterol-lowering effect compared to single alkaloids, which makes them potentially useful for treating hypercholesterolemia (Kou et al., 2016). These results suggest that plants derived compounds show significant lipid-reducing activities and could be used as cholesterol-lowering agents. Table 1; Figure 2 show the mechanisms of phytochemicals regulating the mevalonate pathway.

**TABLE 1:** Phytochemicals regulating the mevalonate pathway.

Phytochemical	Mechanism of action	References
Gallic acid, Catechin, and Epicatechin	•Inhibit 2 (SREBP2) and HMG-CoA	(Shi et al., 2003)
	•Inhibit LDL oxidation	(Ngamukote et al., 2011)
	•Lipid peroxidation	(Millar et al., 2017))
Sesamin, schisandrols A, B and schisandrins B, C	•LXRα/SREBP-1c/FAS/ACC and SREBP2/HMGCR signalling pathways.	(Fukumitsu et al., 2010)
	•Lipid peroxidation	(Glavind et al., 1952)
		(Sevanian and Hochstein, 1985)
Crocin, Chlorogenic acid, Geniposide and Quercetin	•Hypolipidemic effect	Chae et al. (2021)
Curcumin	•Inhibit LDL oxidation	(Ayres et al., 1990)
	•Inhibit the expression of NPC1L1	(Budowski and Markley, 1951)
Ginger	•Inhibit LDL oxidation	Fuhrman et al. (2000)
27-hydroxycholesterol (27-OHC)	•LXR ligand	Warns et al. (2018)
Leonurine	•PPARγ/LXRα signalling pathway	Jiang et al. (2017)
Leoligin	•Inhibition of HMGCR	Scharinger et al. (2016)
Puerarin	•Inhibition of HMGCR	Chung et al. (2008)
	•Activation of the AMPK pathway	
Geraniol	•Inhibition of HMGCR	Galle et al. (2015)
Coptidis	•Unknown	Hadjiphilippou and Ray, (2019)
Coptisine, Palmatine, Epiberberine and Jatrorrhizine	•Unknown	Kou et al. (2016)
Stigmast-4-en-3-one, Cabraleahydroxylactone 3-acetate, and Cabraleahydroxylactone 3-acetate	•Inhibition of LXR	Pham et al. (2021)
β-sitosterol, Campesterol and Stigmasterol	•Activating LXRs/ABCA1	He et al. (2022)
Fucosterol	•Activating LXR	Hoang et al. (2012)

## Cancer and the Mevalonate Pathway

Carcinogenesis is a complex process involving large-scale reprogramming of cell genetic information, signaling mechanisms, structural components, and energy metabolism (Biswas et al., 2012; Teicher et al., 2012). It promotes the existence of a group of malignant disorders that are characterized by deregulated cell proliferation, resistance to apoptosis and amplified cell survival signals and the ability to metastasize to distant organs by invading adjacent tissues (Baba and Câtoi, 2007).

Although a wide range of metabolic pathways are related to the carcinogenic processes and serve as potential targets for the prevention, diagnosis or treatment of cancer, there are relatively few reports on the biosynthesis or metabolism of cholesterol in this process. Carcinogenic effects are typically characterized by the upregulation of cholesterol biosynthesis and uptake, as well as the downregulation or damage caused by cholesterol extrusion. Cholesterol is synthesized by enzymatic reactions referred to as the mevalonate pathway. In addition to cholesterol synthesis, the mevalonate pathway produces isoprenoids that help regulate cell growth (Ortiz et al., 2021). The statins that were tested inhibited the translocation of K-Ras proteins into cell membranes in human pancreatic cancer cell line MiaPaCa-2 cells. All the intermediates in the mevalonate pathway that were tested partially prevented statin inhibition on GFP-K-Ras protein trafficking (Gbelcová et al., 2008; Gbelcová et al., 2017).

There is increasing evidence that cholesterol plays an important role in the progression and development of cancer. Therefore, several strategies have been studied that involve regulatory factors affecting blood and cell cholesterol levels. Statins and biophosphonates can inhibit tumour growth and proliferation by inhibiting the mevalonate pathway (Clendening and Penn, 2012; Thurnher et al., 2012). Ongoing research on key regulators of tumor metabolic pathways is a promising target for anti-tumor drugs, amongst the tumor metabolic pathways, studies have focused on the mevalonate pathway in human malignancies such as leukemia as a driver of oncogenesis and a clinical target (Banker et al., 2004), breast cancer (Siddiqui et al., 2009), ovarian cancer (de Wolf et al., 2017), pancreatic cancer (Mohammed et al., 2012), oesophageal cancer (Zhong et al., 2014) and prostate cancers (Hager et al., 2006). Cholesterol production and posttranslational modifications of Rho GTPases are controlled by the mevalonate pathway, and both are linked to several key aspects of tumor progression (Chen and Hughes-Fulford, 2001; Cruz et al., 2013; Cardama et al., 2017). Proliferation, survival, metastasis, and invasion of tumor cells are all known to be impacted by the mevalonate pathway (Parrales et al., 2018).

Proliferating normal tissues or tumors produce more cholesterol and have a faster rate of cholesterol biosynthesis. Inhibition of phospholipid-remodeling enzyme Lpcat3 drives intestinal stem cells to proliferate by increasing membrane saturation and stimulating cholesterol synthesis. Lpcat3-deficient in hematopoietic cells and mice exhibited normal crypt proliferation when cholesterol synthesis was inhibited pharmacologically (Thomas et al., 2018). By contrast, increasing the cholesterol content in stimulated human intestinal crypts growth, and increasing dietary cholesterol or increasing endogenous cholesterol synthesis through SREBP-2 expression promoted intestinal cell proliferation (Wang et al., 2018a). Additionally, disruption of Lpcat3-dependent cholesterol and phospholipid homeostasis significantly increased tumour formation in min, multiple intestinal neoplasia mice. Wang et al. (Wang et al., 2018a) identified a dietary-responsive phospholipid-cholesterol axis that regulates intestinal stem cell proliferation and tumor development.

Inhibition of cholesterol biosynthesis inhibits cell growth, which further indicates that there is a link between the synthesis of cholesterol, and carcinogenesis; however, molecular mechanisms behind this link remain less understood. Evidence suggests that farnesyl residues from the cholesterol biosynthetic pathway play a critical role in the activation of the G proteins and Ras p21 oncoprotein (Bathaie et al., 2017), which could explain how these two proteins work together. In this way, farnesylation of G proteins and ras p21 oncoproteins further prove that cholesterol biosynthesis has a crucial role in cancer formation. Interestingly, there are mutations in the *ras* gene in several types of cancer; these mutations may increase GTP binding and lead to activated p21. Due to the stimulation of cholesterol biosynthesis pathways in tumours, continued farnesylation will facilitate the activation of p21. Therefore, the cholesterol biosynthesis pathway and ras-p21 can be used as targets for cancer chemoprevention (Bathaie et al., 2017). Mevalonate metabolism can also be inhibited by drugs like bisphosphonates and statins, thus, serving as antitumor agents. A family of statins have been demonstrated capacity to induce apoptosis in a number of cancer cells, which include, colorectal cancer (Chang et al., 2013), breast cancer (Huang et al., 2020a), lung cancer (Ghavami et al., 2010), and ovarian cancer (Martirosyan et al., 2010). Some of these statin-induced anticancer activities are dependent on the genome guardian, p53, even though p53-independent apoptotic pathways have also been reported (Åberg et al., 2008). In many cancer instances, p53 is mutated and it is incapable of suppressing cholesterol-mediated cancer development.

**FIGURE 3:**
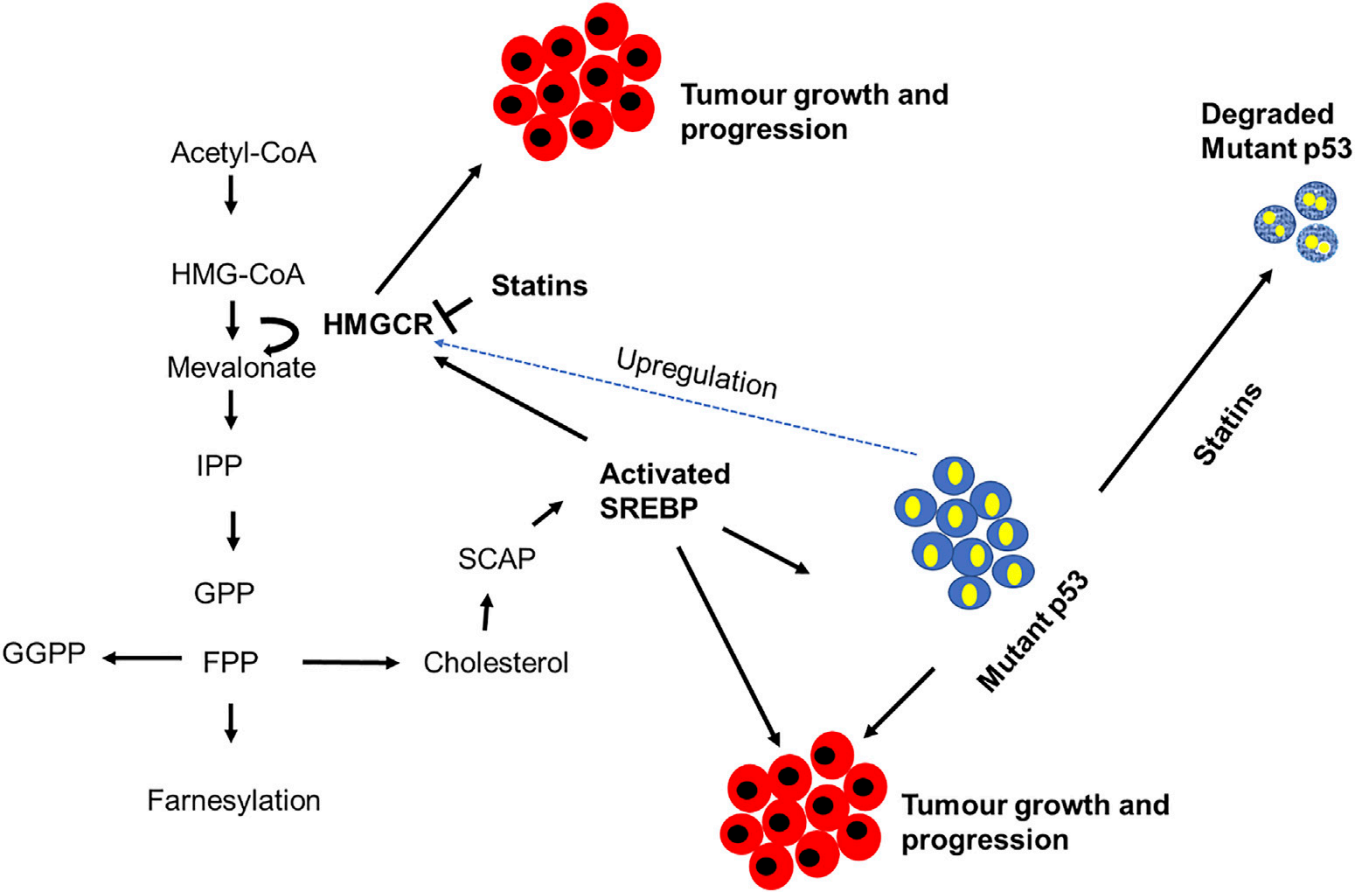
The link between p53 and the mevalonate pathway in cancer cells. This figure illustrates how the mevalonate pathway contributes to tumorigenesis using various enzymes or end products. Mutant-p53 upregulates this pathway by activating sterol regulatory element-binding protein (SREBP), 3-hydroxy-3-methylglutharyl-coenzyme A (HMG-CoA); 3-hydroxy-3-methylglutaryl-CoA reductase (HMGCR); isopentenyl pyrophosphate (IPP); Geranyl pyrophosphate (GPP); farnesyl pyrophosphate (FPP); geranylgeranyl pyrophosphate (GGPP); SREBP cleavage-activating protein (SCAP) [7].

The p53 exists in a mutated form in more than half of human cancers and can significantly up-regulate mevalonate metabolism and protein prenylation in cancer cells (Thurnher et al., 2012). The mevalonate pathway is upregulated in a variety of cancers such as breast cancer (Brown et al., 2016), pancreatic cancer (Mohammed et al., 2012), prostate cancer (Hager et al., 2006), leukemia (Parrales et al., 2018), lung cancer (Wang et al., 2018a) and oesophageal cancer (Lacroix et al., 2019). Quite a few mechanisms have been linked to the deregulation of this pathway, and these include p53 mutation, HMG-CoAR mutation, SREBP cleavage, PKB/Akt activation, decreased AMPK activation and activation of transcription factors such as SREBP and hypoxia-inducible factors (HIF-1). Cancer prevention and/or treatment may be possible by using statins as mevalonate pathway inhibitors through their ability to degrade mutant-p53 (Cerqueira et al., 2016; Iannelli et al., 2018), and interactions with essential cellular functions, such as cell proliferation and differentiation (Hissa and Pontes, 2018). The link between p53, mevalonate pathway and tumor progression are illustrated in Figure 3. Mutant-p53 is shown to bind to SREBP2, which leads to the upregulation of mevalonate pathway enzymes (Parrales et al., 2018). It is enticing to look at new therapeutic targets that target p53 modulation against a number of different cancers, especially those that are affected by deregulated mevalonate pathway.

## Cancers Affected by the Mevalonate Pathway

The Mevalonate pathway is an important regulator of tumor biology and a potential therapeutic target. Several steps in the progression of cancer are controlled by this pathway, including cholesterol production and posttranslational modifications of Rho-GTPases (Göbel et al., 2020b). HMG-CoA reductase, the enzyme responsible for converting HMG-CoA into mevalonate, is the heart of the mevalonate pathway. It has been shown that several tumors exhibit either a deficient feedback control mechanism or increased expression and activity of HMG-CoA reductase (Göbel et al., 2020b). These results insinuate that HMG-CoA reductase may play an essential role in human malignancies. On that note, enzymes involved in the mevalonate pathway such as HMG-CoA reductase, farnesyl diphosphate synthase, squalene synthase and squalene epoxidase are overexpressed and activated in a number of cancers including breast (Siddiqui et al., 2009), melanoma (Kuzu et al., 2016), glioblastoma (Abate et al., 2017), lung cancer (Wang et al., 2018b), ovarian cancer (de Wolf et al., 2017) colorectal (Duncan et al., 2004) and prostate (Hager et al., 2006). Overexpression of mevalonate pathway enzymes is often linked to amplified survival, worse prognosis and clinical outcome, or early recurrence (Göbel et al., 2020b).

Mevalonate is a precursor to cholesterol, which is also necessary for cell proliferation and is formed by HMG-CoA reductase. Cells are arrested in the G1 phase as a result of mevalonate depletion, partly due to impaired activity of cyclin-dependent kinase (CDK) 2 and decreased expression of positive regulators of gap 1 phase (G1) to synthesis phase (S phase) progression. Therefore, inhibiting mevalonate production may be a useful strategy for stopping malignant cells from multiplying. In addition, statins inhibit cholesterol synthesis by blocking HMG-CoA reductase, which is essential for a number of cancer-associated signaling molecules, including those in the Ras/Rho family (Jakobisiak and Golab, 2003; Yang et al., 2020). FDFT1 is lowly expressed in some cancers; however, (Kim et al., 2018; Ha and Lee, 2020; Weng et al., 2020) researchers found that farnesyl diphosphate synthase (FDPS), a key enzyme in isoprenoid biosynthesis, plays a crucial role in maintaining stemness in glioblastomas. Glioblastoma cells were apoptotic when FDPS was knocked down (Brusselmans et al., 2007).

A cholesterol biosynthesis enzyme, squalene synthase promotes lung cancer metastasis by modulating cholesterol biosynthesis and lipid raft formation (Weng et al., 2020). It is imperative to note that squalene synthase knockdown also attenuates proliferation and induces apoptosis in prostate cancer cells (Jun et al., 2021). It has been suggested that squalene epoxidase, another rate-limiting enzyme in cholesterol biosynthesis, may be a proto-oncogene. As paradoxical as it may sound, excess cholesterol reduces squalene epoxidase, and low levels are associated with aggressive colorectal cancer (Escurriol et al., 2009). Through its interaction with the extracellular signal-regulated kinase (ERK) signaling pathway, squalene epoxidase promotes the proliferation, migration, and invasion of lung squamous cell carcinomas cells (Ge et al., 2019). As a possible therapeutic target against cancer, cholesterol metabolism-related enzymes and other related biomolecules have been gaining attention. Mevalonate enzymes involved in various cancer are tabulated in Table 2.

**TABLE 2:** Deregulated mevalonate pathway-linked enzymes in different cancers.

Mediator/Target	Tumour type	References
Mevalonate Kinase	Breast cancer	Brown et al. (2016)
Hydroxymethylglutarate coenzyme-A reductase	Melanoma	Kuzu et al. (2016)
Farnesyl-diphosphate synthase	Glioblastoma	Abate et al. (2017)
Geranylgeranyl diphosphate synthase	Lung adenocarcinoma	Wang et al. (2018b)
Hydroxymethylglutarate coenzyme-A reductase	Ovarian cancer	de Wolf et al. (2017)
Hydroxymethylglutarate coenzyme-A reductase, Farnesyl pyrophosphate synthase, Farnesyltransferase	Colorectal cancer	Duncan et al. (2004)
Farnesyl pyrophosphate synthase	Prostate cancer	Thurnher et al. (2012)
Hydroxymethylglutarate coenzyme-A reductase	Pancreatic cancer	Mohammed et al. (2012)
Hydroxymethylglutarate coenzyme-A reductase	acute myeloid leukemia	Zhong et al. (2014)
Mevalonate diphosphate decarboxylase, Hydroxymethylglutarate coenzyme-A reductase, Acetoacetyl-CoA thiolase 2	Esophageal squamous cell carcinoma	Huang et al. (2020a)
Hydroxymethylglutarate coenzyme-A reductase	Hepatocellular carcinoma	Thrift et al. (2019)

## Effect of Mevalonate on Mutant-p53 and Anticancer Therapeutic Potential

As a consequence of mutations, cancer cells undergo a variety of metabolic transformations, which include activation of oncogenes and inhibition of tumor suppressor genes. A number of oncogenic and tumor suppressor factors are also involved in cholesterol biosynthesis, for example, p53 plays a huge role in the mevalonate pathway (Freed-Pastor et al., 2012). Loss of p53 function has been linked to dysregulated biosynthesis of cholesterol (Chan et al., 2021; Loughran and Emerling, 2022). p53, which is mostly mutated in cancers, blocks SREBP activation, which is required for the activation of genes involved in cholesterol synthesis (Freed-Pastor et al., 2012).

The *ABCA1* cholesterol transporter gene is transcriptionally induced by p53, blocking the activation of SREBP-2, the master transcriptional regulator of this pathway. The existence of p53-dependent downregulation of the mevalonate pathway gene expression in premalignant hepatocytes suggests that p53 is important for the suppression of tumour formation in liver. Even though the molecular mechanism by which ABCA1 increases ER cholesterol levels and, as a consequence, decreases SREBP-2 maturation is not clear, the evidence demonstrates that ABCA1 transcription is one of the mechanisms by which p53 regulates the maturation of SREBP-2 (Moon et al., 2019). Furthermore, p53 is thought to regulate a number of metabolic aspects of cellular metabolism and may contribute to tumour suppression through a variety of metabolic mechanisms and could also influence mevalonate synthesis independently of ABCA1 (Goldstein et al., 2012).

The effect of p53 on mevalonate pathway has been demonstrated in breast tissue (Sorrentino et al., 2014; Ray et al., 2016). Breast cancer cells in a 3D culture model exhibiting a disorganized morphology were reverted to a more normal shape when the mutant-p53 was genetically knocked down or the cholesterol biosynthesis pathway was pharmacologically inhibited (Ray et al., 2016). Furthermore, a wide range of phytochemicals such as crocetin (Ray et al., 2016), quercetin (Yang et al., 2016), curcumin (Yang et al., 2018), gallic acid (Watson et al., 2010), hispidin (Lim et al., 2014) and capsaicin (Jin et al., 2014) have also shown a potential as possible anticancer agents that can be used to restore p53 function in cancer cells. This is due to their success in promoting p53-mediated functions including induction of apoptosis in cancer cells (Ahmed et al., 2016). Curcumin and andrographolide induced p53- and caspase-independent cell death in human neuroblastoma cells by reducing NFκB activity and reducing B-cell lymphoma 2 (Bcl-2) and B-cell lymphoma extra-large (Bcl-xL) expression (Sukumari‐Ramesh et al., 2011). It has been reported that herbal compounds such as α-mangostin and gambogic acid inhibit the p53- Mouse double minute 2 homolog (MDM2) interaction by binding to MDM2 (Hientz et al., 2017). These two compounds showed high binding affinity to hydrophobic MDM2 through the residues Gly58, Asp68, Val75, and Cys77 (Parks et al., 2005).

Treatment with statins reduced mortality in this population-based study. The levels of cellular apoptosis, inhibition of cell growth, and regulation of lipid raft content were significantly higher in mutant-p53 lung cancer cells treated with simvastatin compared to untreated samples. A study conducted on lung cancer cells that had p53 missense mutations also indicated that simvastatin upregulated the caspase-dependent apoptotic pathway, which included mutant-p53 proteolysis, and inhibited motility. Based on these results, statins may be beneficial for lung cancer patients. By blocking the mevalonate pathway in spheroid culture, statins interrupted the biosynthesis of pyrimidine nucleotides and induced oxidative stress and apoptosis in p53-deficient cancer cells (Chou et al., 2019). A further study demonstrated that ubiquinone produced by the mevalonate pathway was necessary for the growth of p53-deficient tumour organoids. In p53-deficient cancer cells, SREBP2, activate the mevalonate pathway and increase ubiquinone levels in the body; ubiquinone is crucial for reducing oxidative stress and supporting pyrimidine nucleotide synthesis (Kaymak et al., 2020).

The phenotypic effects of mutant-p53 on breast tissue architecture can be attributed to statins and sterol biosynthesis intermediates. SREBP transcription factors are responsible for at least some of the association between mutant-p53 and sterol promoters. Further, in human breast tumours, p53 mutations are correlated with highly expressed sterol biosynthesis genes, such as *dependent steroid dehydrogenase-like* (*NSDHL*), *FDFT1*, *MVK* and *transmembrane 7 superfamily member 2* (*TM7S2*). Findings suggest that the mevalonate pathway may offer therapeutic benefits for tumours with p53 mutations (Freed-Pastor et al., 2012). A series of enzymes tightly regulate the mevalonate pathway. HMGCR is the rate-limiting enzyme, and statins can inhibit it; such inhibition has been shown to have antitumor effects in many tumour types. In clinical observations, statin use has been associated with increased liver cancer risk (Ribas et al., 2016).

Cancer cells expressing mutant-p53 grow less rapidly when statins are present. By impairing DNAJA1 DnaJ heat shock protein family (Hsp40) member A1, interaction with mutant-p53, statins or mevalonate kinase knockdown result in a specific reduction of mevalonate-5-phosphate, thus, promoting mutant-p53, ubiquitylation, and degradation. The knockdown of DNAJA1 also leads to the degradation of mutant-p53 by C-terminus of heat shock protein 70 (Hsc-70) interacting protein (CHIP), whereas its overexpression inhibits statin-induced mutant-p53 degradation. DNAJA1 is involved in regulating mutant-p53's fate, and this may provide insights into potential strategies to deplete mutant-p53 through the mevalonate pathway-DNAJA1 axis. Furthermore, this may illuminate the role of statins in modifying the effectiveness of cancer therapy through p53 status (Zhong et al., 2014). Mevalonate pathway dysregulation is caused by p53 mutations, contributing to cancer development. In liver cancer (HCC), the haploid-insufficient tumour suppressor apoptosis-stimulating of p53 protein 2 (ASPP2), an activator of p53, inhibits tumor growth by negatively regulating the mevalonate pathway. In human cancer, ASPP2 is down-regulated, and this is linked to poor prognosis and metastatic spread (Shi et al., 2015). When ASPP2 is downregulated, the expressions of key enzymes in the mevalonate pathway are elevated. ASPP2 binds to SREBP-2 in the nucleus and inhibits the transcription of the target genes of SREBP-2, which include key enzymes in the mevalonate pathway (Liang et al., 2019). The apoptosis stimulated protein of p53-2 (ASPP2) is an enzyme that stimulates p53’s pro-apoptosis function.

The retinoblastoma (Rb) tumour suppressor protein is also considered a modulator of the mevalonate pathway (Hagiwara et al., 2018). In a mouse model of C-cell adenoma, loss of *Rb1* encoding retinoblastoma protein (pRb) improved isoprenylation and activation of NRAS. As a consequence of the deletion of Rb, the transcription factors E2F1 and E2F3, which bind and activate the promoters of numerous prenyltransferase genes, including *farnesyl diphosphate synthase* (*Fdps*) and *sterol regulatory element binding transcription factor 1* (*SREBF1*), become less inhibited. In addition, Rb prevented sterol regulatory element-binding protein 1 (SREBP1) and SREBP2 from promoting the association with the Fdps, indicating that both transcriptional and posttranscriptional effects of Rb are detrimental to the mevalonate pathway (Shamma et al., 2009). The effects of phytochemicals on the function of Rb in cholesterol biosynthesis remain unclear.

The metabolism of cholesterol produces metabolites and necessary membrane components with multiple biological functions. In the tumour microenvironment, the inner and outer parts of the cell signals reprogram cholesterol metabolism, thus promoting tumorigenesis. Cholesterol-derived metabolites play complex roles in supporting cancer progression and overpowering immune responses (Huang et al., 2020b). Various mechanisms that promote deregulation of cholesterol homeostasis stimulate the onset and development of cancer. For that reason, targeting the synthesis of cholesterol and the mevalonate pathway represents a promising therapeutic possibility (Mullen et al., 2016). Cholesterol is one of the requirements for the growth and survival of cancer cells, making it a promising anticancer strategy to reduce intracellular cholesterol biosynthesis. A high level of cholesterol may affect the immune response to cancer and the efficacy of various therapies due to the mevalonate pathway (Assaily et al., 2011).

Although p53 suppresses tumorigenesis, we still lack a complete understanding of how it works. Researchers continue to research p53’s roles in all aspects of cellular metabolism as well as its tumour-suppressing functions (Kou et al., 2016). A mutation within the coding region of the *p53 tumour suppressor* gene, which is one of the mostly altered genes, is able to confer oncogenic properties. There are two gain-of-function mutations in p53, and these include TP53R273H and TP53R280K that impact the activation of the mevalonate pathway through interaction with nuclear SREBP2. This activation of mevalonate pathway genes is necessary and enough for mutant-*p53* to interrupt the normal breast acinar morphology. Furthermore, the expression of a mutant form of p53 in primary breast cancer tissues was linked with the high expression of sterol biosynthesis genes leading to poor prognosis in breast cancer patients (Moon et al., 2019).

In contrast, under glucose-deprived conditions, p53 can inhibit lipid synthesis by promoting the expression of lipin 1 (LPIN1) [122], which prevents SREBPs from binding to the chromatin. In tumours containing these specific gain-of-function mutations in p53, an interaction between the p53 and mevalonate signaling pathways may make the MVA pathway a new therapeutic target. Moon and colleagues (Moon et al., 2019) reported that wild-type *p53* inhibits the genes of the mevalonate pathway by inhibiting SREBP-2 maturation, which is caused by the up-regulation of *ABCA1* transcription promoted by p53. Both wild-type and mutant-p53 interconnect with the master transcriptional regulator, SREBP-2, demonstrating the importance of the mevalonate pathway in cancer. BCA1 reduces total cholesterol levels by exporting excess cholesterol to the plasma membrane (Lawn et al., 1999). The absence of ABCA1 uniquely increases the maturity of SREBP-2 and does not depend on the p53 status.

Mevalonate pathway activation can promote cancer in a variety of ways. Tumor-suppressing and oncogenic pathways are partly dependent on the mevalonate pathway. In the case of cancer cells with abnormal metabolism and growth, mevalonate pathway is up-regulated to provide the necessary building blocks for continued proliferation (Kou et al., 2016). The mevalonate pathway is activated in mutant-p53 cancer cells and allows cell-cycle progression under conditions of low sterol content. It is also possible to detect changes in metabolic flux because of p53-mediated regulation of mevalonate pathway-related genes. Compared with cells containing wild-type p53, mutant-p53 cells showed greater progression to the S phase after sterol starvation, with a significant increase in mature SREBP-2 levels. These results suggest that the inhibition of SREBP-2 maturation by p53 may not be related to the proliferation rate or cell cycle stage. Future research will need to evaluate how pathway rewiring occurs, and which pathway intermediates and by-products contribute to the carcinogenic physiology of mutant-p53 cancer cells. As discussed previously, the chemical structure of most phytochemicals that have biological activity and are useful for pharmaceuticals and cosmeceuticals are mainly composed of polyphenols (Bensinger and Christofk, 2012), having multiple ring structures, a high miscibility in aqueous solutions, and a high molecular weight (Russell, 1992).

## Bioavailability of Phytochemicals

Food compounds and their biological activity have been determined extensively by scientific studies. Unfortunately, these analyses do not suffice to determine the effects of these chemicals on the human body. Several changes occur in the structure of food ingredients during digestion, which can impact their absorption and bioactivity. In many cases, phenolic aglycones are hydrophilic and can be absorbed by diffusion through biological membranes. The majority of polyphenols, however, are glycosidic, which undoubtedly affects their absorption in the intestines. Likewise, oligopeptides can be absorbed via secondary active transport using the hydrogen ion gradient or peptide transporter 1 (PepT1). Food matrix and molecular weight play a big role in the bioavailability of phytochemicals (Karaś et al., 2017). However, phenolic phytochemicals rarely absorb into the body, which limits their bioactivity. They have low water solubility, poor stability, passive diffusion, and active efflux in the gastrointestinal tract, contributing to their low absorption and bioavailability. Pharmaceutical companies use nanoparticle delivery systems extensively to enhance bioactive component absorption (Li et al., 2015).

## Conclusion and Remarks

Based on the evidence given in this review and based on the current understanding of cholesterol metabolism, previous studies have primarily explored the effects of phytochemicals on the regulation of lipid homeostasis from a genetic perspective and discussed the cholesterol-lowering mechanism of phytochemicals as well as the link between tumour suppressor p53 and the mevalonate pathway. In addition to providing cholesterol and substrates for prenylation of a variety of tumor-promoting signaling proteins, the mevalonate pathway is an integral part of cellular homeostasis that has received significant attention in the tumorigenesis literature. Tumor cells require specific metabolic products to ensure their survival, which causes hyperactivation of this pathway. Using traditional herbal medicine ingredients in combination may be beneficial in improving cholesterol levels and preventing obesity-related complications as well as tumor growth. The role of phytochemicals is diversified by its structure-function interaction and can be considered as leads for therapeutic drug design in the future. As a result, these lines may lead to new ways to improve lipid homeostasis. It may prove helpful to identify a role for the mevalonate pathway and responsible driver mutations in the progression of tumors, from initiation to primary tumor growth, through metastatic spread to distant organs, and to secondary outgrowth. The knowledge about the bioavailability and toxicity of phytochemicals must be improved in order to achieve the maximum benefits for the management of cholesterol. High-quality studies with systemic and in-depth analyses are necessary in order to determine the antihyperlipidemic effects of phytochemicals. To ensure hypolipidemic effects, large-scale clinical trials are necessary.
